# Genetic Risk of Autism Spectrum Disorder in a Pakistani Population

**DOI:** 10.3390/genes11101206

**Published:** 2020-10-15

**Authors:** Madiha Khalid, Hashim Raza, Terri M. Driessen, Paul J. Lee, Leon Tejwani, Abdul Sami, Muhammad Nawaz, Shahid Mehmood Baig, Janghoo Lim, Ghazala Kaukab Raja

**Affiliations:** 1 Department of Biochemistry, University Institute of Biochemistry and Biotechnology, PMAS Arid Agriculture University, Rawalpindi 46000, Pakistan; mkmadiha87@gmail.com (M.K.); abdulsami2005@gmail.com (A.S.); 2 Department of Genetics, Yale School of Medicine, New Haven, CT 06510, USA; terri.driessen@gmail.com; 3 Pakistan Institute of Medical Sciences, Islamabad 44000, Pakistan; hraza64@hotmail.com; 4 Interdepartmental Neuroscience Program, Yale School of Medicine, New Haven, CT 06510, USA; jongseo.lee@yale.edu (P.J.L.); leon.tejwani@yale.edu (L.T.); 5 Department of Rheumatology and Inflammation Research, Institute of Medicine, Sahlgrenska Academy, University of Gothenburg, 41346 Gothenburg, Sweden; nawazm.edu@gmail.com; 6 Human Molecular Genetics Laboratory, Health Biotechnology Division, National Institute for Biotechnology and Genetic Engineering (NIBGE), Faisalabad 38000, Pakistan; shahidbaig@nibge.org; 7 Department of Neuroscience, Yale School of Medicine, New Haven, CT 06510, USA; 8 Program in Cellular Neuroscience, Neurodegeneration and Repair, Yale School of Medicine, New Haven, CT 06510, USA; 9 Yale Stem Cell Center, Yale School of Medicine, New Haven, CT 06510, USA

**Keywords:** autism spectrum disorder, genotyping, genetic association, single nucleotide polymorphism, *CNTNAP2*, *ATP2B2*

## Abstract

Autism spectrum disorder (ASD) is a group of complex multifactorial neurodevelopmental and neuropsychiatric disorders in children characterized by impairment of communication and social interaction. Several genes with associated single nucleotide polymorphisms (SNPs) have been identified for ASD in different genetic association studies, meta-analyses, and genome-wide association studies (GWAS). However, associations between different SNPs and ASD vary from population to population. Four SNPs in genes *CNTNAP2, EIF4E, ATP2B2, CACNA1C*, and SNP rs4307059 (which is found between *CDH9* and *CDH10* genes) have been identified and reported as candidate risk factors for ASD. The aim of the present study was, for the first time, to assess the association of SNPs in these genes with ASD in the Pakistani population. PCR-based genotyping was performed using allele-specific primers in 93 ASD and 93 control Pakistani individuals. All genetic associations, genotype frequencies, and allele frequencies were computed as odds’ ratios (ORs) using logistic regression with a threshold of *p* ≤ 0.01 to determine statistical significance. We found that the homozygous genotypes of mutant T alleles of *CNTNAP2* and *ATP2B2* were significantly associated with Pakistani ASD patients in unadjusted ORs (*p* < 0.01), but their significance score was lost in the adjusted model. Other SNPs such as rs4307059, rs17850950 of *EIF4E*, and rs1006737 of *CACNA1C* were not statistically significant. Based on this, we conclude that SNPs are not associated with, or are not the main cause of, autism in the Pakistani population, indicating the involvement of additional players, which need to be investigated in future studies in a large population size. One of the limitations of present study is its small sample size. However, this study, being the first on Pakistani ASD patients, may lay the foundations for future studies in larger samples.

## 1. Introduction

Autism is a complex group of neurodevelopmental disorders, also referred to as autism spectrum disorder (ASD). The term “spectrum” is used to describe the symptoms involving a wide range of skill impairments in ASD children. Some children display milder impairments while others may present more severe forms of ASD [1,2].

ASD is characterized by deficiencies in three main areas of development, which include defects in nonverbal and verbal communication, social interaction, and the presence of multiple repetitive behaviors with limited or unusual interests [3]. The prevalence of ASD has steadily increased over the last few decades. Initially, in the 1970s, ASD was considered a rare disorder, and its prevalence was estimated to be in around 2 of 10,000 children [4]. Towards the end of the 20th century, ASD prevalence began to change, which in 2006 increased to 116.1 per 10,000 children in the United Kingdom [5]. Several factors may contribute to this increase, such as changes in the use of screening tools and diagnostic methods as well as in the application of different epidemiological methods. Beyond the increased awareness among the general population and healthcare professionals contributing to the increased estimated incidence of ASD, it is also possible that other factors have resulted in an unusual increase in ASD occurrence [6].

According to the most recent estimate in the United States, 1 out of 59 children are affected with ASD [7]. Importantly, differences in the occurrence of ASD among different ethnic populations have been noted. For instance, in South Korea, the estimated prevalence of ASD is 2.64% [8] and in around 12 per 10,000 in China [9]. As discrepancies exist concerning the prevalence of ASD among different populations, it is difficult to compare the estimates of ASD prevalence in different regions. This, in part, may be due to the different methods of case identification [6]. ASD is diagnosed more commonly in males than in females, whereby boys are at a 4- to 7-fold higher risk of developing autism compared to girls; however, the reason behind this is still largely unknown [10]. Currently, ASD diagnosis is based on behavioral parameters by identifying the deviation from a typical behavior pattern, but what is considered typical may vary between different cultures. Hence the variability in the extent of deviation from typical behaviors in ASD can be influenced by cultural norms and values [11,12].

ASD is also reported as a complex genetically heritable disorder with a heritability of approximately 50% [13]. Several genetic factors have been found to contribute to the disease. Chromosomal abnormalities or single-gene mutations have been documented in familial and individual autism cases [14]. However, these defined mutations and de novo copy number variants account for only about 10–20% of ASD cases, leaving a high percentage of ASD cases with unknown genetic causes [15,16]. Besides genetic factors, certain environmental factors are also reported to contribute to ASD. Among them is included in utero exposure of offspring to viral or bacterial infection, which may lead to failures in early fetal brain development [17,18]. In addition, the risk of autism due to prenatal infections is most likely dependent on the individual immune status of the mother and fetus. This was confirmed when a substantial association with ASD was found in the allergies and autoimmune disease of the mother [19]. Since placenta serves as the source of hematopoietic stem cells for the fetus, these maternal infections could change the immune status of the fetal immune system as well as fetal brain development [20].

Genetic architecture of ASD is highly heterogeneous. Several genetic association studies and genome-wide association studies (GWAS) of ASD in different populations have identified a number of genes, SNPs and common genetic variants associated with the risk of ASD [21,22,23]. Among them, genetic marker rs4307059, which lies between cadherin 10 *(CDH10)* and cadherin 9 *(CDH9)*, and the SNPs in the gene *ATP2B2* are the major ASD risk markers because of their associations with ASD in different populations. *ATP2B2* encodes plasma membrane calcium-transporting ATPase 2 (PMCA2), which extrudes calcium (Ca^2+^) from the cytosol into the extracellular space in response to increased cytosolic Ca^2+^ concentrations [22,24]. *ATP2B2* is involved in maintaining intracellular calcium homeostasis, and disrupting this balance leads to seizures in ASD [25]. Several pieces of evidence from biochemical and genetic studies have indicated that altered Ca^2+^ homeostasis play a key role in the cascade of signaling events leading to ASD. Furthermore, *ATP2B2* is expressed mainly in cerebellum, along with cerebral cortex, olfactory bulb, and hippocampal formation [26]. *ATP2B2* is located in the human chromosome region 3p25.3. Several studies have linked the region in chromosome 3p25 with ASD [27]. The first evidence of the association between *ATP2B2* and ASD was provided by Carayol J, et al. in a family-based association study [28]. Later on, similar results were replicated in another research in Italy [29]. These researches indicated that *ATP2B2* might be a susceptible candidate gene for ASD [25]. On the other hand, the strongest GWAS and replicated evidence for association of an SNP rs4307059 on 5p14.1 and ASD has been found [30,31]. Recent GWAS from China has also shown a strong association of rs4307059 with ASD [32]. Of particular note, the chromosomal region containing rs4307059 also contains the transcript for the noncoding antisense RNA of the moesin pseudogene 1 (MSNP1), whose overexpression decreases the neurite length in human cells [33].

Furthermore, in European population GWAS studies, the SNPs in Eukaryotic translation initiation factor 4E (*EIF4E)* and the Contactin-associated protein-like 2 (*CNTNAP2)* have been proposed as strong risk candidates for ASD [34,35]. In a eukaryotic translation initiation, *EIF4E* is the rate-limiting component and plays a key role in memory and in learning through its control of translation within the synapse. Increased activity of *EIF4E* causes repetitive behaviors in ASD [36,37]. *CNTNAP2* is the first widely replicated ASD-predisposition gene with the strongest evidence of ASD susceptibility from several independent studies [38,39]. *CNTNAP2* encodes for Contactin Associated Protein-Like 2 (Caspr2), is localized at the juxtaparanodes of myelinated axons, and is thought to be involved in axon differentiation. Caspr2 plays a role as a receptor and cell adhesion molecule in the vertebrate nervous system. The neuronal circuits involved in higher cortical functions are enriched in Caspr2. It plays a major role in language development in ASD and other language-related disorders [40]. Mice lacking *CNTNAP2* show similarity to the core deficits of cognitive and behavioral functions and selective dysregulation of connectivity in integrative prefrontal areas that are seen in ASD patients, signifying its vital role in brain development [41].Genetic variants within another gene, *CACNA1C*, are reported to be linked with psychiatric disorders including ASD, schizophrenia, and bipolar disorder. *CACNA1C* encodes the α 1C subunit (Cav 1.2) of the L-type voltage-dependent calcium channel and calcium influx through such channels is coupled to signaling pathways that stimulate the expression of genes essential for neuronal survival dendritic development, synaptic plasticity, learning, memory formation, and behavior. Rare mutations in *CACNA1C* are known to cause cognitive abnormalities in ASD [42,43,44]. On the basis of previous research and heterogeneity between the results of genetic association studies among different populations, the abovementioned five genes have been selected for the present study.

ASD is well studied in Europe and America but is relatively less understood in the Eastern world [6]. To date, most of genetic association studies on ASD have been primarily carried out in North America, Western Europe, and Australasia. Asia represents the largest portion of world’s population with different ethnic backgrounds and genomic heterogeneity. Therefore, there is a need to explore the genetic markers responsible for ASD in Asian populations. So far, most of the ASD-related genetic association studies have been performed on Chinese, Korean, and Indian populations, but there is no genetic association study reported in the Pakistani population as of yet [45,46,47,48]. There is even no reliable data available regarding the prevalence of ASD in Pakistan [49]. However, according to the estimates of the Pakistan Autism Society, about 350,000 children are suffering from ASD in Pakistan [50].

Examining the genetic variants in Pakistani individuals has been challenging, as individuals with psychiatric disorders do not report their disease due to the possibility of social stigma. This results in an under-reporting of the number of individuals in Pakistan with mental illnesses and prevents patients from receiving care by trained professionals. The present study has addressed this gap in our knowledge by examining five common ASD SNPs among a Pakistani cohort for the first time. This study may not only provide an initial step towards the awareness of ASD among parents and society, but also highlights the underlying genetic causes among Pakistani ASD children.

## 2. Materials and Methods

### 2.1. Procedure and Participants

This study was approved by the Ethics committee of Pir Mehr Ali Shah Arid Agriculture University Rawalpindi and the Pakistan Institute of Medical Sciences (PIMS) Hospital, Islamabad. Written informed consent was obtained from the parents of 93 patients and 93 healthy controls who participated in this study. Cases were recruited from PIMS hospital Islamabad, Pakistan after a consensus diagnosis made by experienced psychiatrists using a combination of the Autism Diagnosis Observation Schedule (ADOS) and the Diagnostic and Statistical Manual of Mental Disorders 5th edition (DSM-5) as the assessment instruments. Clinical phenotype data was collected by asking questions to the parents of children. Healthy controls were recruited from local communities with a simple non-structured interview performed by psychiatrists. Control subjects with a history of mental health or neurological disease, or first-degree relatives suffering from mental health or neurological disease, were excluded from the present study. Healthy controls were drawn from the same geographical areas as patients and were matched to the patient group based on ethnicity. All participants were unrelated Pakistani nationals born and residing in different areas of Pakistan.

### 2.2. DNA Extraction and Quantification

Venous blood was collected in Ethylenediaminetetraacetic acid (EDTA) vacutainers from patients and healthy controls. Genomic DNA was extracted by the standard phenol-chloroform method with few modifications in the protocol [51]. Whole blood (750 μL) and equal amount of red blood cell (RBC) lysis solution (0.32 M Sucrose, 10 mM Tris-HCl pH 7.5, 5 mM MgCl_2_, 0.01% Triton-X) were collected in an Eppendorf tube and incubated at room temperature for five minutes. Then centrifugation was performed at 12,000 revolutions per minute (rpm) for 2 min at 4 °C; 750 μL of RBC lysis solution was added to the pelleted cells after discarding the supernatant. Washing steps were repeated three times until a clear white blood cells (WBC) pellet was obtained. Then, to the pelleted cells, 450 μL of WBC lysis solution (10 mM Tris-HCl pH 7.5, 400 mM NaCl, 2 mM EDTA pH 8.0), 10 μL of 20% sodium dodecyl sulphate (SDS) solution, and 10 μL of Proteinase K (20 mg/μL) were added and incubated overnight at 37 °C. The next day, 300 μL of chloroform-isoamylalcohol solution (24:1) and 300 μL of phenol (pH 7.8) were added to the lysed WBCs and centrifugation was performed at 12,000 rpm for 10 min at 4 °C. The upper aqueous layer was collected in a new clean tube, and 55 μL of 3M sodium acetate solution and 800 μL of chilled isopropanol were added to precipitate the genomic DNA.

The solution was centrifuged for 10 min at 12,000 rpm, the supernatant was discarded, and the pellet of DNA was washed with 250 μL of 70% ethanol. Centrifugation was performed again for 10 min at 12,000 rpm and ethanol was removed by keeping the tubes inverted for 10 min which were then air dried. The DNA pellet was then dissolved in 100 μL of TE (100 mM Tris-HCl pH7.5, 10 mM EDTA pH 8.0) and stored at −20 °C for genotyping. DNA quantification was done using a Nanodrop Spectrophotometer 2000 (Thermo Scientific, Waltham, MA, USA). Each sample was diluted to a final concentration of 10 ng/μL before genotyping.

### 2.3. Genotyping

Genotyping was conducted on SNPs in the genes *ATP2B2, CNTNAP2, CACNA1C, EIF4E* and in the rs4307059 marker, which lies between the *CDH9* and *CDH10* genes at Yale school of medicine, Yale University, Connecticut, USA. IDT oligoanalyzer (IDT Technologies, Coralville, IA, USA) and BatchPrimer 3 (https://wheat.pw.usda.gov/demos/BatchPrimer3/) were used to design allele-specific primers and the primers for Sanger sequencing (Table S1). PCR-based amplification was performed using the reaction mixture with 1× PCR buffer (No. 10342020; Invitrogen, Carlsbad, CA, USA), 1.5 nM of MgCl2, 0.2 mM dNTPs, 0.5 μM primers, 1 unit of Taq DNA polymerase and 20 ng genomic DNA. Amplification was performed with a DNA Engine Peltier Thermal Cycler (Bio-Rad, Hercules, CA, USA) with the following cycling conditions: initial denaturation at 95 °C for 5 min, followed by 30 cycles at 95 °C for 30 s, Tm specific for each primer for 30 s, and 72 °C for 45 s, with a final elongation of 72 °C for 5 min.

Agarose gel (1.5%) was used for running the PCR products. Several randomly selected samples were sent for Sanger sequencing to confirm the specificity of allele-specific primers using allele-flanking primers (Table S1). For sequencing, bands of DNA were cut from the gel followed by DNA extraction using the MinElute gel extraction kit (No. 28604; Qiagen, Hilden, Germany). Finally, the Sanger sequencing was performed at the Keck DNA Sequencing Lab at Yale University, Connecticut, USA and a 4Peaks system was used to visualize electropherograms.

### 2.4. Genotyping Data Analysis

Difference in age and gender between the cases and control groups was determined by *t*-test and χ-squared test in Prism v7 (GraphPad, San Diego, CA, USA). Linkage disequilibrium and Hardy Weinberg equilibrium was calculated by Bioconductor’s R package “genetics” and “HardyWeinberg”, respectively. A bonferroni corrected *p*-value was applied to the Hardy Weinberg *p*-values and all subsequent statistical tests to account for multiple testing of five different SNPs in the same samples. The nominal *p*-value calculated in SAS v9.4 (SAS Institute, Cary, NC, USA) that surpassed the 0.01 Bonferroni-corrected *p*-value was considered significant. This methodology has been employed for GWAS and genetic case-control studies, and has been described in detail in previously published protocol papers [52,53]. The relative risk of SNPs to disease and interaction between gender and each SNP was determined using a multinomial logistic regression model in SAS. Both unadjusted and adjusted *p*-values, odds ratios (ORs), and 95% confidence intervals (95% CI) were calculated for each SNP. Adjusted models included age and gender as covariates. To identify an additive effect of risk alleles from multiple SNPs, the genetic risk scores analysis was performed. Furthermore, a χ-squared test was performed to check any association of clinical variables with SNP genotypes in SPSS v16 (IBM, Chicago, IL, USA).

### 2.5. Protein–Protein Interaction Network

The interaction between the selected proteins ATP2B2, CNTNAP2, CACNA1C, and CDH8 and CDH9 spanning the rs4307059 and other proteins related to ASD was explored using STRING version 10.5 [54]. We considered only high confidence protein–protein interactions obtained from experimental, co-expression, co-occurrence, or database sources. STRING analysis also included 20 primary and 20 secondary interactors along with selected proteins. Identification of ASD-linked KEGG pathways (https://www.genome.jp/kegg/) was also conducted.

## 3. Results

### 3.1. Patient Characteristics and Minor Allele Fequencies

In the present study, we compared 93 autistic individual cases with an equal number of healthy controls from the Pakistani population by genotyping to assess the association of SNPs in *ATP2B2*, *CNTNAP2*, *CACNA1C*, rs4307059, and *EIF4E* with the disease risk. The chromosomal position of each SNP is provided in Supplementary Table S2. The average age and gender of patients and controls are listed in Table 1. We found significant differences between the controls and ASD patients in age and gender (*p*-value < 0.001). These variables were used as covariates in subsequent statistical analysis. All controls passed Hardy-Weinberg equilibrium, and minor allele frequencies in our population were analyzed with globally reported frequencies in Table S2.

### 3.2. Genotyping and Sanger Sequencing

**Figure 1:**
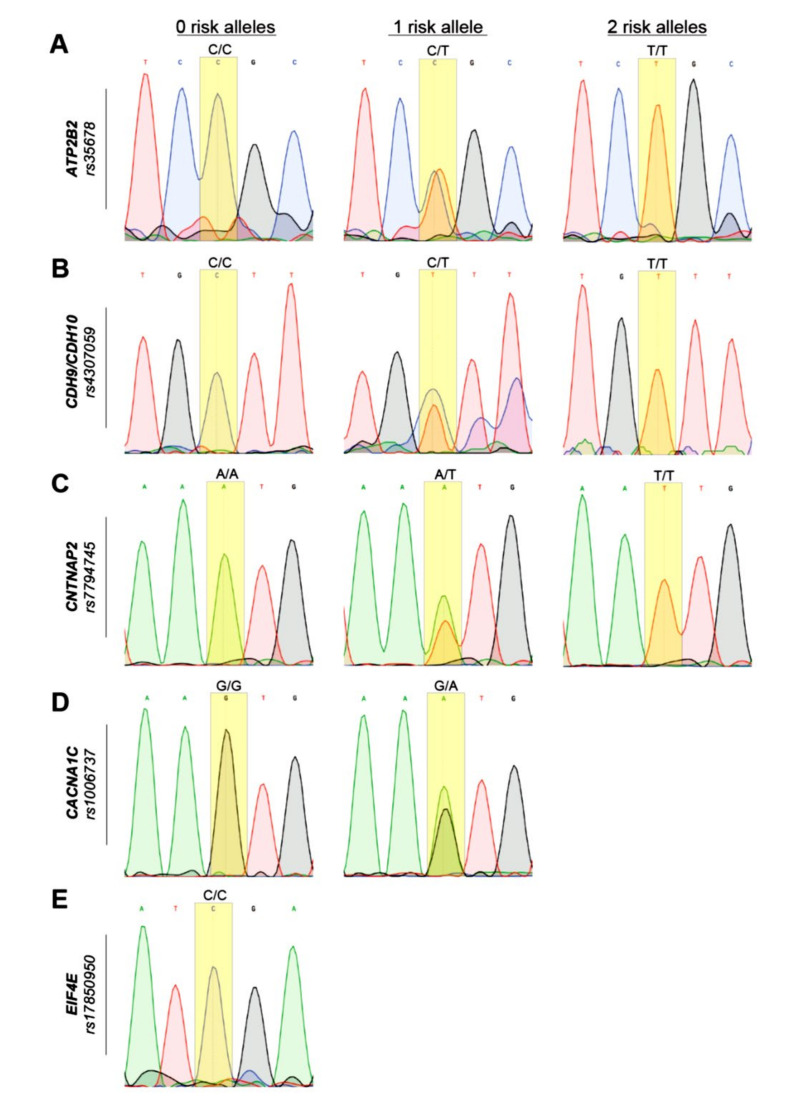
Sanger sequencing of selected samples showing all observed genotypes. Electropherograms of individuals with (**A**) *ATP2B2*/rs35678 C/T, (**B**) Rs4307059 C/T, (**C**) CNTNAP2/rs7794745 A/T, (**D**) CACNA1C/rs1006737 G/A, (**E**) EIF4E/rs17850950 C/T. All three possible genotypes are highlighted.

Genotyping was conducted on SNPs in the genes *ATP2B2*, *CNTNAP2*, *CACNA1C*, *EIF4E*, and in the rs4307059 marker, which lies between the CDH9 and CDH10 genes and the specificity of primers was confirmed by Sanger sequencing. The results of Sanger sequencing are shown in Figure 1. Parts A, B, C, D, and E shows sequencing results of *ATP2B2*/rs35678, Rs4307059, *CNTNAP2/*rs7794745, *CACNA1C*/rs1006737, and *EIF4E/*rs17850950, respectively. The measure of 0 risk refers to the homozygous ancestral genotype, 1 risk refers to the presence of one risk allele in the heterozygous condition and 2 risk refers to the presence of two risk alleles in homozygous condition (Figure 1).

### 3.3. Comparison of Allele Frequencies of Studied Population with Global Allele Frequencies 

In a comparison of minor allele frequencies, the results revealed that minor allele T of *ATP2B2* showed a frequency of 0.57 globally, but in the Pakistani population, it showed the relatively higher frequency of 0.65. On the other hand, the minor allele T of *CNTNAP2* was found with the frequency of 0.40 in our population as compared to 0.51 observed by other populations. The frequency of a minor allele A of *CACNA1C* was 0.20 in our population, with a 0.30 globally estimated frequency, while a minor allele T of rs4307059 was also observed to have a difference in frequency of 0.63 in our population as compared to 0.79 reported globally. Minor allele T of rs17850950 of *EIF4E* showed 0 frequency in our population and globally it also showed the very low frequency of 0.01 (Table S2).

### 3.4. Association Analysis

It has been observed in genotyping results of rs17850950 of *EIF4E* that 100% of cases and controls were homozygous for the C allele. We did not find the risk allele T in any homozygous or heterozygous combination among cases or controls (Table 2). For the SNP rs35678 of *ATP2B2*, 15.1% controls and 3.2% ASD cases were observed as homozygous for the C allele. Observed frequencies of heterozygotes were 50.5% and 51.6% in controls and cases, respectively, while genotypic frequencies of controls and cases were 34.4% and 45.2%, respectively, for the homozygous risk allele genotype (TT). Unadjusted ORs and *p*-values for heterozygotes were 4.76 and 0.01, respectively, while for the homozygous risk allele genotypes, unadjusted ORs were 6.12 with a *p*-value of 0.007 (Table 2). After adjusting for age and gender, ORs for heterozygous and homozygous risk allele genotypes were 0.42 and 1.37 with non-significant *p*-values of 0.52 and 0.81, respectively (Table 2).

For SNP rs7794745 of *CNTNAP2*, we observed in 40.9% controls and 30.1% autistic cases homozygous for A allele, while 50.5% controls and 48.4% cases were heterozygous. For the homozygous risk allele combination (TT), 8.6% of controls and 21.5% of cases were observed. The homozygous genotype showed unadjusted OR of 3.39 (unadjusted *p*-value 0.01, Table 2), while the adjusted OR was 2.98 (*p*-adj. 0.83). In rs1006737 of *CACNA1C*, 62.4% of controls were homozygous for the G allele as compared to 55.9% cases, while 37.6% controls and 44.1% cases were heterozygotes (GA), carrying a single copy of the risk allele A. The observed unadjusted OR for the heterozygous genotype was 1.3 (*p*-value 0.37); however, the adjusted OR was 1.41 with a *p*-value of 0.58 (Table 2).

For the SNP rs4307059, we found 50.5% of controls as compared to 48.4% of cases with one risk allele T in the heterozygous genotype CT. In the homozygous risk allele combination TT, 36.6% of controls as compared to 40.9% of cases were observed. Both genotypes showed unadjusted ORs of 1.14 and 1.34 with *p*-values of 0.77 and 0.54, respectively. After adjusting the *p*-value with age and gender, ORs were 0.26 (*p*-value 0.319) and 0.05 (*p*-value 0.05), respectively.

In terms of minor allele frequencies and their association with ASD, our results indicated that the minor allele T of *ATP2B2* and T of *CNTNAP2* were found with unadjusted and adjusted odds of 1.04 (*p*-value 0.88), 0.29 (*p*-value 0.11) and 1.68 (*p*-value 0.01), 0.86 (*p*-value 0.79), respectively. Minor alleles A of *CACNA1C* and T of rs4307059 showed unadjusted ORs of 1.21 (*p*-value 0.44) and 1.16 (*p*-value 0.5) respectively; however, adjusted ORs were 1.21 (*p*-value 0.44) and 0.72 (*p*-value 0.61), respectively (Table 3). Interaction of gender and SNPs also revealed non-significant *p*-values (Table S3).

### 3.5. Risk Score Analysis

In a risk score analysis, no individual was found with a 0 risk allele and therefore, 1 risk allele was taken as a reference. The presence of 2 risk alleles was found with an adjusted OR of 2.17 (*p*-value 0.77) (Table 4). Similarly, the presence of 3 and 4 risk alleles was found with ORs of 3.53 (*p* 0.63) and 3.16 (*p*-value 0.66), respectively. Presence of 5 and more than 5 risk alleles together showed an OR of 2.11 and *p*-value 0.78.

### 3.6. Protein–Protein Interactions and Pathway Analysis

**Figure 2:**
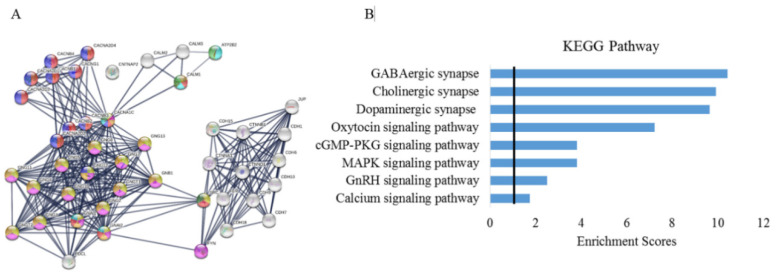
Protein–protein interactions and enrichment analysis for ATP2B2, CNTNAP2, CACNA1C, CDH9, and CDH10 and their interacting partners. (**A**) Using STRING analysis, all forty proteins that interact with ATP2B2, CNTNAP2, CACNA1C, CDH9, and CDH10 via either primary or secondary interactions were plotted. The confidence level of the interactions is represented by the width of the edges connecting the proteins. (**B**) Enrichment analysis for KEGG pathways among the forty proteins that interact with ATP2B2, CNTNAP2, CACNA1C, CDH9, and CDH10. *X*-axis represents the enrichment score, which is the -log FDR-adjusted *p*-value. The black line at 1.3 represents an FDR-adjusted *p*-value and the pathways that surpass 1.3 are significantly over-represented among our proteins of interest.

The STRING analysis to evaluate the interaction of ATP2B2, CNTNAP2, CACNA1C, CDH9, and CDH10 directly or through their primary or secondary partners indicated that they do not interact directly but through primary or secondary interactors at a high confidence except for CNTNAP2, which interacts neither directly nor through any interactors (Figure 2A). Proteins with their interacting partners are shown in Table S4.

Some of the predicted functional partners were identified which include proteins involved in calcium channels (CACNA2D2, CACNA2D3, CACNB2, CACNB3, CACNB1, CACNB4), calcium-dependent cell adhesion proteins (CDH6, CDH7, CDH15, CDH18), and G-protein (GNB1) (Figure 2A, Table S5). Furthermore, some of the identified KEGG pathways by enrichment analysis were found to be associated with the oxytocin signaling pathway, MAPK signaling pathway, calcium signaling pathway, GABAergic synapse, GnRH signaling pathway, Cholinergic synapse, and the cGMP-PKG signaling pathway (Figure 2B).

### 3.7. Association between SNPs and Autistic-Like Traits

The association between 21 clinical variables of ASD was estimated with selected SNPs and our results indicated that rs35678 of *ATP2B2* was found to be associated with olfactory symptoms and poor/limited understanding, with a *p*-value of 0.04 and 0.02, respectively (Table 5). rs7794745 of *CNTNAP2* was associated with a lack of self-care skills and aggressive behavior, with a *p*-value of 0.03 and 0.009, respectively.rs1006737 of *CACNA1C* was found to be associated with aggressive behavior with a *p*-value of 0.01, while rs4307059 was associated with unusual noise-producing behavior and poor/limited understanding with *p*-values of 0.04 and 0.03, respectively. All of these associations become statistically non-significant after Bonferroni correction, except for aggressive behavior association with *CNTNAP2* and *CACNA1C*, with *p*-values of 0.009 and 0.01, respectively (Table 5).

## 4. Discussion

ASD is a form of complex and severe developmental disorder, with strong genetic foundations [13]. In this present study, we investigated the association between *EIF4E*, *ATP2B2*, *CNTNAP2*, *CACNA1C*, and Rs4307059 polymorphisms and ASD in the Pakistani population. Comparing the minor allele frequencies of studied SNPs in our population with globally reported allele frequencies showed the differences indicating genetic heterogeneity in different ethnicities.

The genotyping results of our research indicated that *EIF4E* did not show any type of association; however, previous genome-wide linkage studies in ASD patients have linked the region containing the *EIF4E* locus on chromosome 4q with ASD as a regulation of *EIF4E* activity is known to play a key role in learning and memory through its control of translation within the synapse [55,56,57,58]. In our study, we did not find even a single individual showing minor allele T, which may be due to the fact that this minor allele was found in very low frequency, i.e., 0.01, even in other worldwide populations (1000 Genome).

Previous studies showed a significant association of SNP rs4307059, which is found between the CDH9 and CDH10 gene with ASD [22,59], however no association with ASD was found in the present study as both unadjusted and adjusted *p*-values were non-significant. These results are inconsistent with a previous study on the Italian population, which showed a significant association of rs4307059 with ASD [29]. Various independent GWAS done by using individuals from European and Caucasian ancestry, which reported rs4307059 as a novel ASD associated region [30,60]. Similar results were observed for *CACNA1C* in the present study, although several other independent studies reported the involvement and the association of the specific SNP rs1006737 in *CACNA1C* with psychiatric disorders in European, Danish, and Spanish populations [61,62,63]. However, in our study, we did not find any association of risk allele A of rs1006737 of *CACNA1C* with ASD. Consistent results with the present study were also reported in the Chinese Han population in which rs1003767 was not associated with ASD risk [64]. Allelic expression imbalance was also found for this SNP as no homozygotes for risk alleles (AA) were found among cases and controls.

In the case of SNP rs35678 of *ATP2B2*, an obvious difference between genotypic frequencies of controls (34.4%) and cases (45.2%) for homozygous risk allele genotype (TT) was observed and unadjusted *p*-values were significant for both heterozygous and homozygous risk allele genotypes, but after adjusting for age and gender, it was no longer statistically significant. Similar results were observed in terms of SNP rs7794745 of *CNTNAP2*, when for the homozygous risk allele combination (TT), a significant difference between genotypic frequencies of controls (8.6%) and cases (21.5%) were observed. The homozygous genotype was found to be significantly associated with disease with odds of 3.39 (unadjusted *p* 0.01) but this association did not remain significant after adjusting with covariates (*p*-adj. 0.83). Likewise, in terms of allele frequencies and their association with ASD, only risk allele T of *CNTNAP2* was found to be significantly associated in the unadjusted model, but remained no longer significant in the adjusted model. This lost significance in most of the SNPs may be due to the unequal numbers of male and female patients, as well as the large difference between ages of controls and cases (Table 1). However, if we looked upon the interaction of gender with SNPs, as almost 73% of individuals who participated in this study were male, no significant interaction between any of the four SNPs and gender was observed (Table S3), indicating that gender had no effect on the association between any SNP and disease.

Although risk alleles of both *ATP2B2* and *CNTNAP2* did not show a significant association with ASD in adjusted models, we found a trend of association of studied SNPs with some behavioral phenotypes of ASD. *ATP2B2* showed a trend of association towards a limited understanding and sensory behavior although later on, after Bonferroni correction, significance was lost but this trend of association was strengthened by previous findings, which showed plasma membrane calcium-transporting ATPase 2 (PMCA2) encoded by *ATP2B2* are expressed in brain and sensory systems at a particularly high level and in the developing human brain. Similarly, *CNTNAP2* was found to be associated with a lack of self-care skills and with aggressive behavior. Significant association of *CNTNAP2* and aggressive behavior persisted even after Bonferroni correction.

Additionally, messenger RNA (mRNA) of *CNTNAP2* is reported to be significantly enriched in the temporal and frontal lobes, as well as in the frontal cortex and in striatal circuits of the adult brain [65]. These regions support speech, language learning, and other forms of implicit learning, further strengthening a role of *CNTNAP2* in cognition and language, which are the major affected areas in ASD [66,67]. *CACNA1C* has previously been implicated to be involved in anxiety, cognition, fear conditioning, and depressive phenotypes [68,69]. We also found a significant association of *CACNA1C* with aggressive behavior, with a *p*-value of 0.01.

In polygenic risk score analysis, no combined risk effect of studied SNPs was observed in our study, which is also obvious from STRING analysis, as none of the protein interact directly with each other and also no primary or secondary interacting protein was found to be previously reported in ASD. However, several KEGG pathways were found to be reported early in the case of ASD like oxytocin (OXT), calcium signaling pathways, and GABAergic function. In the hypothalamus, OXT is the biological basis of trust, social recognition, and bonding. It plays major roles in the modulation of social behaviors with a focus on social bonding, recognition, and communication [70]. Calcium signaling pathway disturbance may contribute greatly to the underlying molecular mechanism of ASD [71,72,73]. Furthermore, the characteristic ASD phenotype is often associated with either a loss or a gain of the GABAergic function. Dysfunction of GABAergic signaling mediates ASD-like stereotypes in the majority of animal models of ASD [74]. GABA-mediated calcium signaling regulates a variety of developmental processes from cell proliferation, and therefore it is not unanticipated that some forms of neuro-developmental disorders including ASD showed alterations of GABAergic signaling and impairment of the excitatory/inhibitory balance in selective neuronal circuits [75]. In the brain of ASD patients, insulin-signaling pathways and pathological involvement of cholinergic nuclei and altered expression of acetylcholine receptors, particularly nicotinic acetylcholine receptors, have also been reported [76,77,78,79].

The present study has some limitations, which include the small sample size, as well as the significant difference between age and gender between cases and controls (Table 1). Lack of awareness and misconceptions about psychiatric disorders among the Pakistani population pose a problem to correctly diagnosing and collecting blood/DNA samples for genetic testing. In recognition of these limitations, appropriate statistical analyses accounting for gender and age as covariates were conducted to ensure that there was no effect on statistical results. However, despite these limitations, this study may serve as an initial step to set the foundation for future studies utilizing larger samples from the Pakistani population.

## 5. Conclusions

The present study provided some trend of association of studied SNPs to the etiology of ASD in the Pakistani population. Homozygous risk allele genotypes of *ATP2B2* and *CNTNAP2* were strongly associated with ASD in unadjusted models. In terms of risk allele association, risk allele T of *CNTNAP2* was significantly associated with ASD in an unadjusted model. Significance was lost in the adjusted model, which may be due to the difference in ages of cases and controls. All studied SNPs also showed some trend of association with clinical phenotypes of ASD, whereas *CNTNAP2* and *CACNA1C* showed a significant association with the aggressive behavior of ASD patients. This study will serve as an initial study of the Pakistani population and further association studies in larger samples and functional research are needed.

## Figures and Tables

**Table 1:** Mean age and gender frequency among control and autism spectrum disorder (ASD) patients.

	Controls	ASD	*p*-Value
Total Samples	93	93	data
Mean Age ± SD	39.98 ± 1.87	8.409 ± 0.5784	<0.001 ^a^
Median	40	7	
Interquartile Range	26.5	5.5	
Total Number (Percentage) Males	41 (44.09%)	68 (73.12%)	<0.001 ^b^
Total Number (Percentage) Females	52 (55.91%)	25 (26.88%)	

^a^ Calculated using an independent *t*-test. ^b^ Calculated using a χ-square test.

**Table 2:** Genotypic frequencies of studied SNPs in control and ASD patients.

Genotype	Controls (*n* = 93)	Cases (*n* = 93)	OR (95% CI) *p*-Value	Adjusted OR (95% CI) *p*-Value
*ATP2B2/*rs35678 (C/T)				
CC	14 (15.1%)	3 (3.2%)	Reference	
CT	47 (50.5%)	48 (51.6%)	*4.76 (1.28–17.66) 0.01	0.42 (0.02–6.23) 0.52
TT	32 (34.4%)	42 (45.2%)	*6.12 (1.62–23.13) 0.007	1.37 (0.09–19.88) 0.81
*CNTNAP2/*rs7794745 (A/T)				
AA	38 (40.9%)	28 (30.1%)	Reference	
AT	47 (50.5%)	45 (48.4%)	1.29 (0.68–2.45) 0.42	0.98 (0.26–3.68) 0.977
TT	8 (8.6%)	20 (21.5%)	*3.39 (1.30–8.81) 0.01	2.98 (0.28–31.16) 0.361
*CACNA1C*/rs1006737 (G/A)				
GG	58 (62.4%)	52 (55.9%)	Reference	
GA	35 (37.6%)	41 (44.1%)	1.30 (0.72–2.34) 0.37	1.41 (0.40–4.92) 0.584
*(CDH9/CDH10)/*rs4307059 (C/T)				
CC	12 (12.9%)	10 (10.8%)	Reference	
CT	47 (50.5)	45 (48.4)	1.14 (0.45–2.92) 0.77	0.26 (0.01–3.62) 0.319
TT	34 (36.6)	38 (40.9)	1.34 (0.51–3.49) 0.54	0.05 (0.002–1.02) 0.05
*EIF4E*/rs17850950				
CC	93 (100%)	93 (100%)	--	--
CT	0	0	--	--
TT	0	0	--	--

SNPs: Single nucleotide polymorphisms. The adjusted odds ratio (OR), 95% confidence interval (CI), and *p*-value were calculated after adjusting for gender and age as covariates. * Unadjusted significant *p*-values are in bold.

**Table 3:** Allele frequencies of studied SNPs in control and ASD patients.

Genes/SNPs IDs	Alleles	Controls (%)	Cases (%)	OR (95%CI) *p*-Value	Adjusted OR (95%CI) *p*-Value
*ATP2B2/*rs35678	T	60	71	1.04 (0.59–1.86) 0.88	0.29 (0.06–1.38) 0.11
	C	40	29		
*CNTNAP2/*rs7794745	T	34	46	*1.68 (1.09–*2.61) 0.017	0.86 (0.28–2.65) 0.79
	A	66	54		
*CACNA1C*/rs1006737	A	19	22	1.21 (0.73–2.02) 0.44	1.21 (0.73–2.02) 0.44
	G	81	78		
*(CDH9/CDH10)/*rs4307059	T	62	65	1.16 (0.75–1.80) 0.5	0.72 (0.20–2.58) 0.61
	C	38	35		

The adjusted OR, 95% CI, and *p*-value were calculated after adjusting for gender and age as covariates. * Unadjusted significant *p*-values are bold

**Table 4:** Polygenic risk score between ASD cases and controls.

Number of Risk Alleles	Controls (*n* = 93)	Cases (*n* = 93)	OR (95% CI) *p*-Value	Adjusted OR (95% CI) *p*-Value
1	6 (6.5%)	1 (1.1%)	Reference	
2	15 (16.1%)	8 (8.6%)	3.19 (0.326–31.391) 0.31	2.175 (0.010–463.87) 0.776
3	27 (29.03%)	25 (26.9%)	5.55 (0.624–49.38) 0.124	3.53 (0.019–650.02) 0.634
4	24 (25.8%)	22 (23.7%)	5.49 (0.613–49.32) 0.127	3.16 (0.016–611.30) 0.667
5+	21 (22.6%)	37 (39.7%)	10.56 (1.19–93.77) 0.034	2.114 (0.011–40.7.04) 0.780

The adjusted OR, 95% CI, and *p*-value were calculated after adjusting for gender and age as covariates.

**Table 5:** Association of clinical variables of ASD with genotypes.

Clinical Phenotype	Classification	*ATP2B2* rs35678				*CNTNAP2* rs7794745				*CACNA1C* rs1006737			rs4307059			
		CC	CT	TT	*p*-Value	GG	GA	AA	*p*-Value	GG	GA	*p*-Value	CC	CT	TT	*p*-Value
Licking	Yes	2	29	23	0.824	17	26	11	0.923	26	28	0.076	4	27	23	0.471
	No	1	19	19		11	19	9		26	23		6	18	15	
Hand functioning	Ok	2	45	40	0.150	25	43	19	0.545	47	40	0.162	10	42	35	0.662
	Poor	1	3	2		3	2	1		5	1		0	3	3	
Self-care skills	Fully dependent	2	29	25	0.983	19	30	7	0.031	32	24	0.721	8	28	20	0.527
	Needs help	1	17	16		7	14	13		19	15		2	15	17	
	Good	0	2	1		2	1	0		1	2		0	2	1	
Vision problems	Yes	0	4	1	0.420	0	3	2	0.275	1	4	0.096	1	1	3	0.412
	No	3	44	41		28	42	18		51	37		9	44	35	
Smells everything	Yes	0	15	22	0.045	12	21	4	0.118	18	19	0.251	3	20	14	0.623
	No	3	33	20		16	24	16		34	22		7	25	24	
Looks closely from eye corner	Yes	1	34	29	0.396	18	34	12	0.378	33	31	0.209	6	33	25	0.621
	No	2	14	13		10	11	8		19	10		4	12	13	
Cover ear in noise	Yes	0	28	21	0.130	12	25	12	0.435	26	23	0.559	5	28	16	0.185
	No	3	20	21		16	20	8		26	18		5	17	22	
Rocking and swaying	Yes	3	40	33	0.596	25	36	15	0.413	46	30	0.058	7	41	28	0.074
	No	0	8	9		3	9	5		6	11		3	4	10	
Repetitive behaviors	Yes	3	44	39	0.862	26	41	19	0.857	46	40	0.099	10	43	33	0.206
	No	0	4	3		2	4	1		6	1		0	2	5	
Likes circular moving objects	Yes	1	21	24	0.381	14	24	8	0.610	22	24	0.120	6	23	17	0.659
	No	2	27	18		14	21	12		30	17		4	22	21	
Reciprocates smiles	Yes	2	14	12	0.554	4	15	9	0.209	15	13	0.760	3	15	10	0.748
	Rarely	0	18	13		11	14	6		19	12		4	16	11	
	No	1	16	17		13	16	5		18	16		3	14	17	
Eye contact	Good	2	15	16	0.744	13	15	5	0.485	20	13	0.616	5	13	15	0.666
	Poor	1	31	25		15	28	14		31	26		5	30	22	
	No	0	2	1		0	2	1		1	3		0	2	1	
Unusual noise	Yes	1	27	20	0.582	14	24	10	0.950	27	21	0.946	2	22	24	0.046
	No	2	21	22		14	21	10		25	20		8	23	14	
Echolalia	Yes	0	18	15	0.420	7	18	8	0.382	18	15	0.844	1	17	15	0.201
	No	3	30	27		21	27	12		34	26		9	28	23	
Sense of being praised	Yes	1	13	7	0.450	7	10	4	0.917	9	12	0.171	0	10	11	0.149
	No	2	35	35		21	35	16		43	29		10	35	27	
Aggressive	Yes	0	18	21	0.159	14	12	13	0.009	16	23	0.014	4	17	18	0.672
	No	3	30	21		14	33	7		36	18		6	28	20	
Speech	No Speech	2	21	12	0.249	13	16	6	0.592	21	14	0.920	4	18	13	0.969
	Able to make short sentences	0	13	15		7	14	7		15	13		4	12	12	
	Can string few words	0	8	8		4	8	4		9	7		1	9	6	
	Only a few single words	1	3	7		4	6	1		6	5		1	5	5	
	Normal	0	3	0		0	1	2		1	2		0	1	2	
Idiosyncratic language	Yes	0	6	8	0.522	2	8	4	0.365	7	7	0.629	2	7	5	0.858
	No	3	42	34		26	37	16		45	34		8	38	33	
Understanding (Cognitive)	Good	1	4	0	0.020	1	3	1	0.664	3	2	0.349	0	3	2	0.034
	Limited	0	11	18		6	16	7		13	16		5	7	17	
	Poor	2	33	24		21	26	12		36	23		5	35	19	
Shared enjoyments with parents	Yes	0	5	3	0.742	2	2	4	0.112	3	5	0.273	0	3	5	0.340
	No	3	43	39		26	43	16		49	36		10	42	33	
Follow instructions	Yes	0	6	5	0.976	2	4	5	0.07	8	3	0.08	4	12	14	0.810
	Very simple	1	15	14		7	14	9		12	18		1	5	5	
	No	2	27	23		19	27	6		32	20		5	28	19	

*p*-values were calculated using χ-squared tests. *p*-values that pass the Bonferroni *p*-value threshold of 0.01 are highlighted.

## Supplementary Materials

The following are available online at https://www.mdpi.com/2073-4425/11/10/1206/s1, Table S1: List of Primers used for genotyping and Sanger sequencing, Table S2: Genes/SNPs ID and their position on chromosomes. Minor allele frequency in studied population and in other worldwide populations, Table S3: Interaction of studied SNPs with Gender, Table S4: Proteins at node 1 and their interacting partners at node 2, Table S5: Gene annotation and Ensemble IDs of Nodes.

## Abbreviations

ASD	Autism spectrum disorder
SNP	Single nucleotide polymorphisms
GWAS	Genome wide association studies
OR	Odds’ ratio
CNTNAP2	Contactin Association Protein-like 2
CACNA1C	L-type voltage-dependent calcium channel
PCR	Polymerase chain reaction
DSM	Diagnostic and Statistical Manual
ADOS	Autism Diagnosis Observation Schedule
RBC	Red blood cells
WBC	White blood cells
KEGG	Kyoto encyclopedia of genes and genomes
