# Supplementary material for: Genetic Risk of Autism Spectrum Disorder in a Pakistani Population
Source: Genes (Basel). 2020 Oct 15;11(10):1206. doi: 10.3390/genes11101206 (PMC7602870; doi:10.3390/genes11101206)
Supplement: Supplementary file 1 [file genes-11-01206-s001.pdf]

Supplementary Tables

Supplementary Table 1: List of Primers used for genotyping and Sanger sequencing

Gene/SNP IDs	Primer sequence 5'-3'
***ATP2B2*/rs35678**	
Forward	GCTGTTCCTTAGCAGCCACAGGAGTGG
Reverse	GAGCGGGTGTCAGGGAGAGAAGG
Allele specific Reverse(T allele)	AGGACGTGGAGGAGATCGACCACACA
Allele specific Reverse(C allele)	AGGACGTGGAGGAGATCGACCACACG
***CNTNAP2*/rs7794745**	
Forward	TACAGTGATTCATATTTCAGGCA
Reverse	CTTTAGTGGTGGTATGTGCTTAGCT
Allele specific Reverse(T allele)	ACTGATTTTGACACTTAGTCTTATTAA
Allele specific Reverse(A allele)	ACTGATTTTGACACTTAGTCTTATTAT
***CACNA1C*/rs1006737**	
Forward	GTCCATCTCTCCCACATGCTG
Reverse	TGTGGACCAGCAATCAGTCAC
Allele specific Forward(G allele)	CATTCCATCTCAGCCCGGAG
Allele specific Reverse(A allele)	GAGGTCT CCG GCT CTG AAA ACA T
***(CDH9* and *CDH10)/* rs4307059**	
Forward	GCTGTATCTCACTGCATGTCATTTGC
Reverse	CCTAATTGCAGTATGATTGATTCTCC
Allele specific Reverse(T allele)	GCATGGAAAATATCCTGGTTACATGGAA
Allele specific Reverse(C allele)	GCATGGAAAATATCCTGGTTACATGGAG
***EIF4E*/ rs17850950**	
Reverse	GTTGGTAGAGGGACGGGACATTC
Allele specific Forward(T allele)	TTACTGTCTCTAGCCAAAAGCGGTT
Allele specific Forward(C allele)	TTACTGTCTCTAGCCAAAAGCGGTC

Table S2: Genes/SNPs ID and their position on chromosomes. Minor allele frequency in studied population and in other worldwide populations.

Gene/SNPs IDs	Major/Minor allele	Chromosome/Position	Global MAF (1000 Genome)	MAF in Current Study Population
***ATP2B2/* rs35678**	C/T	3p25.3/10338239	0.57	0.65
***CNTNAP2/* rs7794745**	A/T	7q35-q36.1/146792514	0.51	0.40
***CACNA1C/* rs1006737**	G/A	12p13.33/2236129	0.30	0.20
**(between *CDH9* and *CDH10* genes)/rs4307049**	C/T	5p14.1/ 25967594	0.79	0.63
***EIF4e/*rs17850950**	C/T	4q23/ 98887099	0.01	0

Table S3: Interaction of studied SNPs with Gender

Genotype	Gender Interaction p-value
***ATP2B2/* rs35678** (C/T)	
CC	Reference
CT	0.97
TT	0.96
***CNTNAP2/* rs7794745** (A/T)	
AA	Reference
AT	0.68
TT	0.93
***CACNA1C/* rs1006737** (G/A)	
GG	Reference
GA	0.15
(CDH9/CDH10)/ rs4307059 (C/T)	
CC	Reference
CT	0.66
TT	0.74

Table S4: Proteins at node 1 and their interacting partners at node 2

node1	node2	node1	node2	node1	node2
ATP2B2	CALM1	CDH7	CTNNA1	GNG2	CACNA2D2
ATP2B2	CALM3	CDH7	CTNNB1	GNG2	CACNB2
CACNA1C	CACNA2D1	CDH7	CTNND1	GNG2	CACNB3
CACNA1C	CACNA2D2	CDH7	JUP	GNG2	GNAI1
CACNA1C	CACNA2D3	CDH9	CDH1	GNG2	GNAI2
CACNA1C	CACNA2D4	CDH9	CDH10	GNG2	GNB1
CACNA1C	CACNB1	CDH9	CDH15	GNG2	GNG10
CACNA1C	CACNB2	CDH9	CDH18	GNG2	GNG11
CACNA1C	CACNB3	CDH9	CDH6	GNG2	GNG12
CACNA1C	CACNB4	CDH9	CDH7	GNG2	GNG13
CACNA1C	CACNG1	CDH9	CTNNA1	GNG2	GNG3
CACNA1C	CALM1	CDH9	CTNNB1	GNG2	GNG4
CACNA1C	CALM2	CDH9	CTNND1	GNG2	GNG5
CACNA1C	CALM3	CDH9	JUP	GNG2	GNG7
CACNA1C	GNAI1	CTNNA1	CDH1	GNG2	GNG8
CACNA1C	GNAI2	CTNNA1	CDH10	GNG2	GNGT1
CACNA1C	GNB1	CTNNA1	CDH15	GNG2	GNGT2
CACNA1C	GNG10	CTNNA1	CDH18	GNG2	PDCL
CACNA1C	GNG11	CTNNA1	CDH6	GNG2	SRC
CACNA1C	GNG12	CTNNA1	CDH7	GNG3	CACNA1C
CACNA1C	GNG13	CTNNA1	CDH9	GNG3	CACNA2D2
CACNA1C	GNG2	CTNNA1	CTNNB1	GNG3	CACNB2
CACNA1C	GNG3	CTNNA1	CTNND1	GNG3	CACNB3
CACNA1C	GNG4	CTNNA1	FER	GNG3	GNAI1
CACNA1C	GNG5	CTNNA1	FYN	GNG3	GNAI2
CACNA1C	GNG7	CTNNA1	JUP	GNG3	GNB1
CACNA1C	GNG8	CTNNA1	SRC	GNG3	GNG10
CACNA1C	GNGT1	CTNNB1	CDH1	GNG3	GNG11
CACNA1C	GNGT2	CTNNB1	CDH10	GNG3	GNG12
CACNA2D1	CACNA1C	CTNNB1	CDH15	GNG3	GNG13
CACNA2D1	CACNA2D4	CTNNB1	CDH18	GNG3	GNG2
CACNA2D1	CACNB1	CTNNB1	CDH6	GNG3	GNG4
CACNA2D1	CACNB2	CTNNB1	CDH7	GNG3	GNG5
CACNA2D1	CACNB3	CTNNB1	CDH9	GNG3	GNG7
CACNA2D1	CACNB4	CTNNB1	CTNNA1	GNG3	GNG8
CACNA2D1	CACNG1	CTNNB1	CTNND1	GNG3	GNGT1
CACNA2D2	CACNA1C	CTNNB1	FER	GNG3	GNGT2
CACNA2D2	CACNB1	CTNNB1	FYN	GNG3	PDCL
CACNA2D2	CACNB2	CTNNB1	JUP	GNG4	CACNA1C
CACNA2D2	CACNB3	CTNNB1	SRC	GNG4	CACNA2D2
CACNA2D2	CACNB4	CTNND1	CDH1	GNG4	CACNB2
CACNA2D2	CACNG1	CTNND1	CDH10	GNG4	CACNB3
CACNA2D2	GNAI1	CTNND1	CDH15	GNG4	GNAI1
CACNA2D2	GNAI2	CTNND1	CDH18	GNG4	GNAI2
CACNA2D2	GNB1	CTNND1	CDH6	GNG4	GNB1
CACNA2D2	GNG10	CTNND1	CDH7	GNG4	GNG10
CACNA2D2	GNG11	CTNND1	CDH9	GNG4	GNG11
CACNA2D2	GNG12	CTNND1	CTNNA1	GNG4	GNG12
CACNA2D2	GNG13	CTNND1	CTNNB1	GNG4	GNG13
CACNA2D2	GNG2	CTNND1	FER	GNG4	GNG2
CACNA2D2	GNG3	CTNND1	FYN	GNG4	GNG3

CACNA2D2	GNG4	CTNND1	JUP	GNG4	GNG5
CACNA2D2	GNG5	CTNND1	SRC	GNG4	GNG7
CACNA2D2	GNG7	FER	CDH1	GNG4	GNG8
CACNA2D2	GNG8	FER	CTNNA1	GNG4	GNGT1
CACNA2D2	GNGT1	FER	CTNNB1	GNG4	GNGT2
CACNA2D2	GNGT2	FER	CTNND1	GNG4	PDCL
CACNA2D3	CACNA1C	FER	JUP	GNG5	CACNA1C
CACNA2D3	CACNB1	FYN	CDH1	GNG5	CACNA2D2
CACNA2D3	CACNB2	FYN	CTNNA1	GNG5	CACNB2
CACNA2D3	CACNB3	FYN	CTNNB1	GNG5	CACNB3
CACNA2D3	CACNB4	FYN	CTNND1	GNG5	GNAI1
CACNA2D3	CACNG1	FYN	GNAI2	GNG5	GNAI2
CACNA2D4	CACNA1C	FYN	JUP	GNG5	GNB1
CACNA2D4	CACNA2D1	FYN	SRC	GNG5	GNG10
CACNA2D4	CACNB1	GNAI1	CACNA1C	GNG5	GNG11
CACNA2D4	CACNB2	GNAI1	CACNA2D2	GNG5	GNG12
CACNA2D4	CACNB3	GNAI1	CACNB2	GNG5	GNG13
CACNA2D4	CACNB4	GNAI1	CACNB3	GNG5	GNG2
CACNA2D4	CACNG1	GNAI1	GNAI2	GNG5	GNG3
CACNB1	CACNA1C	GNAI1	GNB1	GNG5	GNG4
CACNB1	CACNA2D1	GNAI1	GNG10	GNG5	GNG7
CACNB1	CACNA2D2	GNAI1	GNG11	GNG5	GNG8
CACNB1	CACNA2D3	GNAI1	GNG12	GNG5	GNGT1
CACNB1	CACNA2D4	GNAI1	GNG13	GNG5	GNGT2
CACNB1	CACNB2	GNAI1	GNG2	GNG5	PDCL
CACNB1	CACNB3	GNAI1	GNG3	GNG7	CACNA1C
CACNB1	CACNG1	GNAI1	GNG4	GNG7	CACNA2D2
CACNB2	CACNA1C	GNAI1	GNG5	GNG7	CACNB2
CACNB2	CACNA2D1	GNAI1	GNG7	GNG7	CACNB3
CACNB2	CACNA2D2	GNAI1	GNG8	GNG7	GNAI1
CACNB2	CACNA2D3	GNAI1	GNGT1	GNG7	GNAI2
CACNB2	CACNA2D4	GNAI1	GNGT2	GNG7	GNB1
CACNB2	CACNB1	GNAI1	PDCL	GNG7	GNG10
CACNB2	CACNB3	GNAI1	SRC	GNG7	GNG11
CACNB2	CACNB4	GNAI2	CACNA1C	GNG7	GNG12
CACNB2	CACNG1	GNAI2	CACNA2D2	GNG7	GNG13
CACNB2	GNAI1	GNAI2	CACNB2	GNG7	GNG2
CACNB2	GNAI2	GNAI2	CACNB3	GNG7	GNG3
CACNB2	GNB1	GNAI2	FYN	GNG7	GNG4
CACNB2	GNG10	GNAI2	GNAI1	GNG7	GNG5
CACNB2	GNG11	GNAI2	GNB1	GNG7	GNG8
CACNB2	GNG12	GNAI2	GNG10	GNG7	GNGT1
CACNB2	GNG13	GNAI2	GNG11	GNG7	GNGT2
CACNB2	GNG2	GNAI2	GNG12	GNG7	PDCL
CACNB2	GNG3	GNAI2	GNG13	GNG8	CACNA1C
CACNB2	GNG4	GNAI2	GNG2	GNG8	CACNA2D2
CACNB2	GNG5	GNAI2	GNG3	GNG8	CACNB2
CACNB2	GNG7	GNAI2	GNG4	GNG8	CACNB3
CACNB2	GNG8	GNAI2	GNG5	GNG8	GNAI1
CACNB2	GNGT1	GNAI2	GNG7	GNG8	GNAI2
CACNB2	GNGT2	GNAI2	GNG8	GNG8	GNB1
CACNB3	CACNA1C	GNAI2	GNGT1	GNG8	GNG10
CACNB3	CACNA2D1	GNAI2	GNGT2	GNG8	GNG11

CACNB3	CACNA2D2	GNAI2	PDCL	GNG8	GNG12
CACNB3	CACNA2D3	GNAI2	SRC	GNG8	GNG13
CACNB3	CACNA2D4	GNB1	CACNA1C	GNG8	GNG2
CACNB3	CACNB1	GNB1	CACNA2D2	GNG8	GNG3
CACNB3	CACNB2	GNB1	CACNB2	GNG8	GNG4
CACNB3	CACNB4	GNB1	CACNB3	GNG8	GNG5
CACNB3	CACNG1	GNB1	GNAI1	GNG8	GNG7
CACNB3	GNAI1	GNB1	GNAI2	GNG8	GNGT1
CACNB3	GNAI2	GNB1	GNG10	GNG8	GNGT2
CACNB3	GNB1	GNB1	GNG11	GNG8	PDCL
CACNB3	GNG10	GNB1	GNG12	GNGT1	CACNA1C
CACNB3	GNG11	GNB1	GNG13	GNGT1	CACNA2D2
CACNB3	GNG12	GNB1	GNG2	GNGT1	CACNB2
CACNB3	GNG13	GNB1	GNG3	GNGT1	CACNB3
CACNB3	GNG2	GNB1	GNG4	GNGT1	GNAI1
CACNB3	GNG3	GNB1	GNG5	GNGT1	GNAI2
CACNB3	GNG4	GNB1	GNG7	GNGT1	GNB1
CACNB3	GNG5	GNB1	GNG8	GNGT1	GNG10
CACNB3	GNG7	GNB1	GNGT1	GNGT1	GNG11
CACNB3	GNG8	GNB1	GNGT2	GNGT1	GNG12
CACNB3	GNGT1	GNB1	PDCL	GNGT1	GNG13
CACNB3	GNGT2	GNB1	SRC	GNGT1	GNG2
CACNB4	CACNA1C	GNG10	CACNA1C	GNGT1	GNG3
CACNB4	CACNA2D1	GNG10	CACNA2D2	GNGT1	GNG4
CACNB4	CACNA2D2	GNG10	CACNB2	GNGT1	GNG5
CACNB4	CACNA2D3	GNG10	CACNB3	GNGT1	GNG7
CACNB4	CACNA2D4	GNG10	GNAI1	GNGT1	GNG8
CACNB4	CACNB2	GNG10	GNAI2	GNGT1	GNGT2
CACNB4	CACNB3	GNG10	GNB1	GNGT1	PDCL
CACNB4	CACNG1	GNG10	GNG11	GNGT1	SRC
CACNG1	CACNA1C	GNG10	GNG12	GNGT2	CACNA1C
CACNG1	CACNA2D1	GNG10	GNG13	GNGT2	CACNA2D2
CACNG1	CACNA2D2	GNG10	GNG2	GNGT2	CACNB2
CACNG1	CACNA2D3	GNG10	GNG3	GNGT2	CACNB3
CACNG1	CACNA2D4	GNG10	GNG4	GNGT2	GNAI1
CACNG1	CACNB1	GNG10	GNG5	GNGT2	GNAI2
CACNG1	CACNB2	GNG10	GNG7	GNGT2	GNB1
CACNG1	CACNB3	GNG10	GNG8	GNGT2	GNG10
CACNG1	CACNB4	GNG10	GNGT1	GNGT2	GNG11
CALM1	ATP2B2	GNG10	GNGT2	GNGT2	GNG12
CALM1	CACNA1C	GNG10	PDCL	GNGT2	GNG13
CALM1	CALM2	GNG11	CACNA1C	GNGT2	GNG2
CALM1	CALM3	GNG11	CACNA2D2	GNGT2	GNG3
CALM2	CACNA1C	GNG11	CACNB2	GNGT2	GNG4
CALM2	CALM1	GNG11	CACNB3	GNGT2	GNG5
CALM2	CALM3	GNG11	GNAI1	GNGT2	GNG7
CALM3	ATP2B2	GNG11	GNAI2	GNGT2	GNG8
CALM3	CACNA1C	GNG11	GNB1	GNGT2	GNGT1
CALM3	CALM1	GNG11	GNG10	GNGT2	PDCL
CALM3	CALM2	GNG11	GNG12	JUP	CDH1
CDH1	CDH10	GNG11	GNG13	JUP	CDH10
CDH1	CDH15	GNG11	GNG2	JUP	CDH15
CDH1	CDH18	GNG11	GNG3	JUP	CDH18

CDH1	CDH6	GNG11	GNG4	JUP	CDH6
CDH1	CDH7	GNG11	GNG5	JUP	CDH7
CDH1	CDH9	GNG11	GNG7	JUP	CDH9
CDH1	CTNNA1	GNG11	GNG8	JUP	CTNNA1
CDH1	CTNNB1	GNG11	GNGT1	JUP	CTNNB1
CDH1	CTNND1	GNG11	GNGT2	JUP	CTNND1
CDH1	FER	GNG11	PDCL	JUP	FER
CDH1	FYN	GNG12	CACNA1C	JUP	FYN
CDH1	JUP	GNG12	CACNA2D2	JUP	SRC
CDH1	SRC	GNG12	CACNB2	PDCL	GNAI1
CDH10	CDH1	GNG12	CACNB3	PDCL	GNAI2
CDH10	CDH15	GNG12	GNAI1	PDCL	GNB1
CDH10	CDH18	GNG12	GNAI2	PDCL	GNG10
CDH10	CDH6	GNG12	GNB1	PDCL	GNG11
CDH10	CDH7	GNG12	GNG10	PDCL	GNG12
CDH10	CDH9	GNG12	GNG11	PDCL	GNG13
CDH10	CTNNA1	GNG12	GNG13	PDCL	GNG2
CDH10	CTNNB1	GNG12	GNG2	PDCL	GNG3
CDH10	CTNND1	GNG12	GNG3	PDCL	GNG4
CDH10	JUP	GNG12	GNG4	PDCL	GNG5
CDH15	CDH1	GNG12	GNG5	PDCL	GNG7
CDH15	CDH10	GNG12	GNG7	PDCL	GNG8
CDH15	CDH18	GNG12	GNG8	PDCL	GNGT1
CDH15	CDH6	GNG12	GNGT1	PDCL	GNGT2
CDH15	CDH7	GNG12	GNGT2	SRC	CDH1
CDH15	CDH9	GNG12	PDCL	SRC	CTNNA1
CDH15	CTNNA1	GNG13	CACNA1C	SRC	CTNNB1
CDH15	CTNNB1	GNG13	CACNA2D2	SRC	CTNND1
CDH15	CTNND1	GNG13	CACNB2	SRC	FYN
CDH15	JUP	GNG13	CACNB3	SRC	GNAI1
CDH18	CDH1	GNG13	GNAI1	SRC	GNAI2
CDH18	CDH10	GNG13	GNAI2	SRC	GNB1
CDH18	CDH15	GNG13	GNB1	SRC	GNG2
CDH18	CDH6	GNG13	GNG10	SRC	GNGT1
CDH18	CDH7	GNG13	GNG11	SRC	JUP
CDH18	CDH9	GNG13	GNG12		
CDH18	CTNNA1	GNG13	GNG2		
CDH18	CTNNB1	GNG13	GNG3		
CDH18	CTNND1	GNG13	GNG4		
CDH18	JUP	GNG13	GNG5		
CDH6	CDH1	GNG13	GNG7		
CDH6	CDH10	GNG13	GNG8		
CDH6	CDH15	GNG13	GNGT1		
CDH6	CDH18	GNG13	GNGT2		
CDH7	CDH6	GNG13	PDCL		
CDH7	CDH9	GNG2	CACNA1C		

Table S5: Gene annotation and Ensemble IDs of Nodes

Nodes	Ensemble IDs	Annotation
ATP2B2	ENSP00000353414	Plasma membrane calcium-transporting ATPase 2;
CACNA1C	ENSP00000266376	Calcium channel, voltage-dependent, L type, alpha 1C subunit
CACNA2D1	ENSP00000349320	Voltage-dependent calcium channel subunit alpha-2/delta-1
CACNA2D2	ENSP00000418081	Voltage-dependent calcium channel subunit alpha-2/delta-2
CACNA2D3	ENSP00000419101	Voltage-dependent calcium channel subunit alpha-2/delta-3
CACNA2D4	ENSP00000372169	Voltage-dependent calcium channel subunit alpha-2/delta-4
CACNB1	ENSP00000377840	Calcium channel, voltage-dependent, beta 1 subunit
CACNB2	ENSP00000320025	Calcium channel, voltage-dependent, beta 2 subunit
CACNB3	ENSP00000301050	Calcium channel, voltage-dependent, beta 3 subunit
CACNB4	ENSP00000438949	Calcium channel, voltage-dependent, beta 4 subunit
CACNG1	ENSP00000226021	Voltage-dependent calcium channel gamma-1 subunit
CALM1	ENSP00000349467	Calmodulin 1
CALM2	ENSP00000272298	Calmodulin 2
CALM3	ENSP00000291295	Calmodulin 3
CDH1	ENSP00000261769	Cadherin 1
CDH10	ENSP00000264463	Cadherin10
CDH15	ENSP00000289746	Cadherin15
CDH18	ENSP00000425093	Cadherin18
CDH6	ENSP00000265071	Cadherin 6
CDH7	ENSP00000381058	Cadherin 7
CDH9	ENSP00000231021	Cadherin 9
CTNNA1	ENSP00000304669	Catenin alpha-1
CTNNB1	ENSP00000344456	Catenin beta-1
CTNND1	ENSP00000382004	Catenin delta-1
FER	ENSP00000281092	Tyrosine-protein kinase Fer
FYN	ENSP00000346671	Tyrosine-protein kinase Fyn
GNAI1	ENSP00000343027	Guanine nucleotide-binding protein G(i) subunit alpha-1
GNAI2	ENSP00000312999	Guanine nucleotide-binding protein G(i) subunit alpha-2
GNB1	ENSP00000481878	Guanine nucleotide-binding protein G(I)/G(S)/G(T) subunit beta-1
GNG10	ENSP00000363411	Guanine nucleotide-binding protein G(I)/G(S)/G(O) subunit gamma-10
GNG11	ENSP00000248564	Guanine nucleotide-binding protein G(I)/G(S)/G(O) subunit gamma-11
GNG12	ENSP00000360021	Guanine nucleotide-binding protein G(I)/G(S)/G(O) subunit gamma-12
GNG13	ENSP00000248150	Guanine nucleotide-binding protein G(I)/G(S)/G(O) subunit gamma-13
GNG2	ENSP00000334448	Guanine nucleotide-binding protein G(I)/G(S)/G(O) subunit gamma-2
GNG3	ENSP00000294117	Guanine nucleotide-binding protein G(I)/G(S)/G(O) subunit gamma-3
GNG4	ENSP00000375727	Guanine nucleotide-binding protein G(I)/G(S)/G(O) subunit gamma-4
GNG5	ENSP00000359675	Guanine nucleotide-binding protein G(I)/G(S)/G(O) subunit gamma-5
GNG7	ENSP00000371594	Guanine nucleotide-binding protein G(I)/G(S)/G(O) subunit gamma-7
GNG8	ENSP00000300873	Guanine nucleotide-binding protein G(I)/G(S)/G(O) subunit gamma-8
GNGT1	ENSP00000248572	Guanine nucleotide-binding protein G(T) subunit gamma-T1
GNGT2	ENSP00000426022	Guanine nucleotide-binding protein G(T) subunit gamma-T2
JUP	ENSP00000377508	Junction plakoglobin
PDCL	ENSP00000259467	Phosducin-like protein
SRC	ENSP00000362680	Proto-oncogene tyrosine-protein kinase Src
